# Prognostic value of C-reactive protein-albumin-lymphocyte (CALLY) score and CRP-albumin ratio on mortality in patients diagnosed with pulmonary thromboembolism

**DOI:** 10.3389/fmed.2026.1944670

**Published:** 2026-10-26

**Authors:** Ahmet Mert Aydemir

**Affiliations:** Elazig Fethi Sekin City Hospital, Emergency Medicine, Elazığ, Türkiye

**Keywords:** CALLY, C-reactive protein-to-albumin ratio, mortality, pulmonary thromboembolism, risk stratification

## Abstract

**Background:**

Acute pulmonary embolism (PE) requires rapid risk stratification to predict early mortality. We aimed to evaluate the prognostic value of the C-reactive protein-albumin-lymphocyte (CALLY) index and C-reactive protein-to-albumin ratio (CAR) in predicting in-hospital mortality among patients with PE.

**Methods:**

This retrospective, single-center Study Included 399 Patients Diagnosed with Acute PE Between 2021 and 2025. the Clinical Characteristics and Baseline Laboratory Parameters Were Analyzed. Receiver Operating Characteristic (ROC) Curves Were Used to Determine the Optimal Biomarker cut-offs. Univariate and Multivariate Logistic Regression Models Were Constructed to Evaluate the Independent Predictors of Mortality

**Results:**

Non-survivors (n=88,22.0%) had significantly lower CALLY indices (0.71 vs. 1.56,p<0.001) and higher CAR values (2.37 vs. 0.93,p<0.001) than survivors (n=311,78.0%). The AUC was 0.655 for CALLY (<1.17 cut-off; 69.1% sensitivity, 57.7% specificity, 86.4% NPV) and 0.643 for CAR (>3.28 cut-off; 42.0% sensitivity, 83.2% specificity, 83.2% NPV) (DeLong test, p=0.582). In multivariate models adjusted for age, sex, and serum creatinine, both a low CALLY index (<1.17; aOR=2.54,95%CI:1.46−4.50,p=0.001) and a high CAR (>3.28; aOR=3.02,95%CI:1.66−5.50,p<0.001) served as independent predictors of in-hospital mortality.

**Conclusion:**

The baseline CALLY index is a simple, cost-effective, and readily Accessible Biomarker That Independently Predicts in-hospital mortality in Patients with Acute PE. A CALLY Index <1.17 Provides Moderate Sensitivity for Early Emergency Department Risk Stratification, though its overall discriminative performance is modest

## Introduction

Pulmonary thromboembolism is a high-mortality clinical condition requiring rapid diagnosis and risk stratification, and the clinical need for practical biomarkers that can be used to predict prognosis remains current (1). In this context, the CALLY score, which integrates C-reactive protein, albumin, and lymphocyte levels, simultaneously reflects both the inflammatory response and the patient's nutritional status, and thus holds strong biomarker potential for predicting early mortality risk in patients with pulmonary embolism (2). Considering the decisive effect of systemic inflammatory processes on the pathophysiology of pulmonary embolism, the success of this score in predicting mortality may optimize clinical risk stratification (3, 4). In particular, the dynamic interaction between acute-phase reactants and nutritional parameters provides more objective data for detecting clinical deterioration in patients than standard scoring systems (5, 6). Among the components forming the basis of the CALLY score, CRP reflects systemic inflammation and a hypercoagulable state, albumin represents nutritional reserve, and lymphocytes indicate the body's immune response capacity (7, 8). The combination of these biomarkers, especially for assessing the systemic immune-inflammatory response that arises after pulmonary embolism (9), provides a more comprehensive risk analysis than the CRP/albumin ratio alone in terms of its prognostic value (10). Previously validated for malignancy-associated inflammation in oncological processes and for the assessment of atherosclerotic risk in cardiovascular pathologies, this score offers a unique perspective in acute pulmonary embolism, reflecting both ischemia due to vascular occlusion and the subsequent systemic cytokine storm (11). This study aimed to examine the effect of the CALLY index, determined from blood samples taken during the initial assessment of patients monitored in the emergency department, on mortality.

## Materials and methods

This study was conducted retrospectively after obtaining approval from the ethics committee. Patients who presented to the Emergency Department of Elazığ Fethi Sekin City Hospital and were diagnosed with pulmonary embolism were evaluated in this study. After identifying patients in the Hospital Information Management System between 01.01.2021 and 31.12.2025, clinical evaluations of the patients and findings from pulmonary CT angiography were examined. Following these evaluations, patients over 18 years of age who were diagnosed with PE were included in the study, while those without PE on clinical evaluation and imaging, patients under 18 years of age, and those with incomplete data were excluded from the study. After identifying the patients who met the inclusion and exclusion criteria, their demographic data, mortality status, mortality duration, and biochemical results were recorded. The CALLY score was calculated based on the biochemical results of the patients.

$$\mathrm{CALLY}\;\mathrm{Score}=\frac{\mathrm{Albumin}\left(\frac{g}{L}\right)*\mathrm{Lymphocytes}\left(\frac{10^{9}}{L}\right)}{\mathrm{CRP}\;\left(\frac{\mathrm{mg}}{L}\right)}$$

By analyzing the created database, we determined the association between the CALLY score and mortality.

This retrospective, single-center study was conducted at the Emergency Department of Elazığ Fethi Sekin City Hospital. We initially screened all patients diagnosed with pulmonary embolism through the Hospital Information Management System between January 1, 2021, and December 31, 2025. Acute pulmonary embolism was confirmed in all included patients via computed tomography pulmonary angiography (CTPA). For patients with multiple admissions during the study period, only the initial presentation was included to prevent duplicate entries, and cases of chronic pulmonary embolism were systematically excluded. Patients under 18 years of age, those without definitive PE confirmation on clinical evaluation and imaging, and patients with incomplete electronic medical records or missing laboratory data were excluded from the final analysis Figure 1.

**Figure 1:**
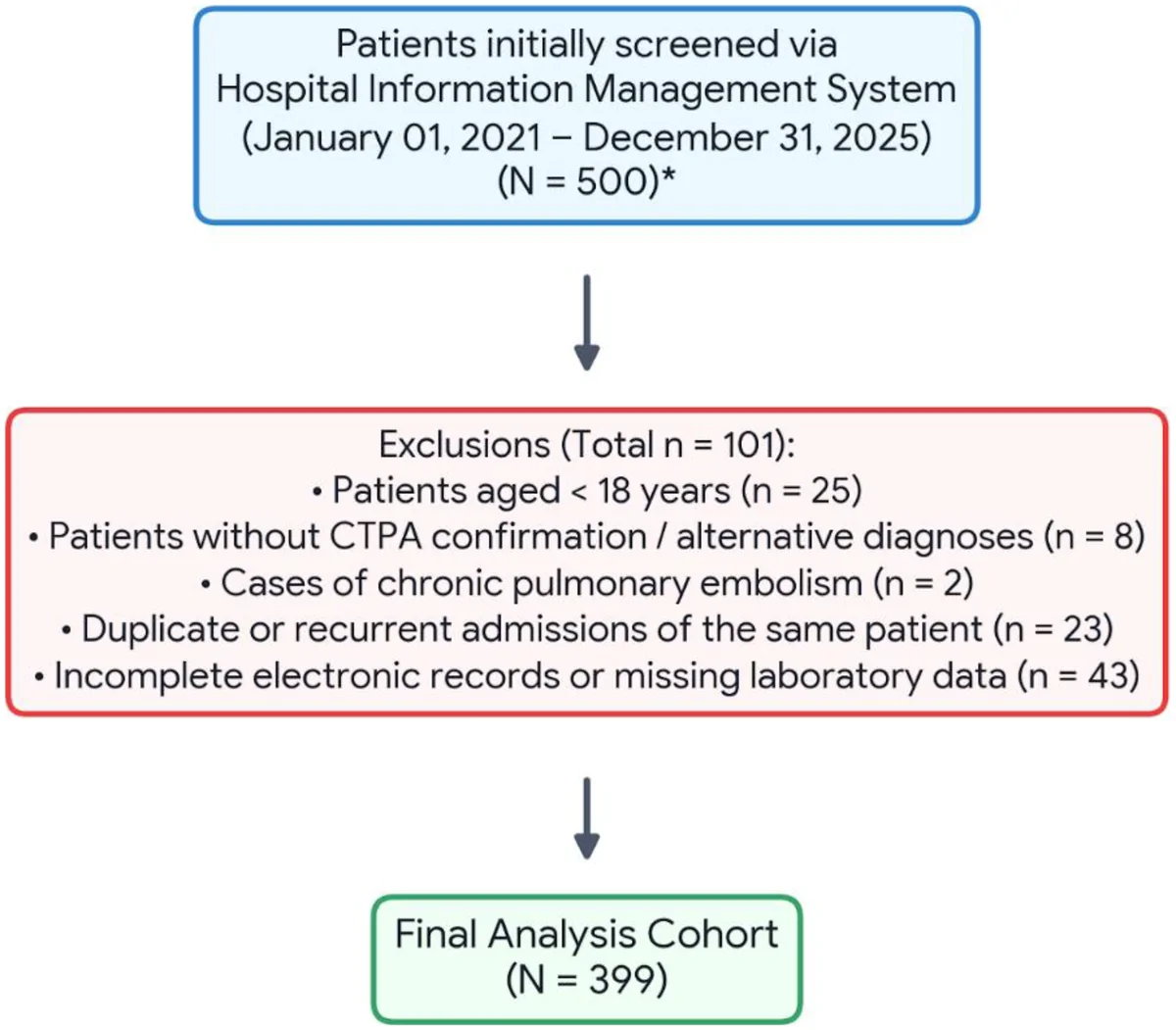
Patient flow diagram.

### Statistical analysis

Statistical analyses were performed using R Statistical Software (version 4.3.2; R Foundation for Statistical Computing, Vienna, Austria) within the RStudio/Posit Cloud integrated development environment. The primary endpoint of this study was in-hospital mortality.

Descriptive statistics were presented as medians and interquartile ranges (IQR:25th−75thpercentiles) for continuous variables based on non-normal data distributions assessed via the Shapiro–Wilk test, and as absolute numbers and percentages (n,%) for categorical variables. Comparisons between survivors (live group) and non-survivors (ex group) were conducted using the Mann–Whitney U test for continuous variables and the Pearson Chi-square test or Fisher's exact test for categorical variables.

Receiver Operating Characteristic (ROC) curve analysis was performed using the pROC package to evaluate the discriminative capacity of the CALLY index and CAR for in-hospital mortality. The Area Under the Curve (AUC) and 95% Confidence Intervals (95% CI) were calculated. Optimal cut-off thresholds were determined using the maximum Youden's J statistic (Sensitivity + Specificity - 1), and corresponding sensitivity, specificity, positive predictive value (PPV), and negative predictive value (NPV) were reported. Pairwise comparisons of the AUCs were conducted using DeLong's test.

Univariable and multivariable logistic regression analyses were performed to identify independent predictors of in-hospital mortality. To avoid collinearity between closely related composite indices, two separate multivariable models were constructed: Model 1 evaluated the CALLY index (both categorical <1.17 and continuous log-transformed) adjusted for age, sex, and serum creatinine, while Model 2 evaluated the CAR (both categorical >3.28 and continuous log-transformed) adjusted for the same covariates. Odds Ratios (OR) and adjusted Odds Ratios (aOR) with their corresponding 95% CIs were reported. All statistical tests were two-tailed, and a *p*-value <0.05 was considered statistically significant

Univariate and multivariate logistic regression analyses were performed using the gtsummary and broom.helpers packages to identify independent predictors of in-hospital mortality. To assess the independent prognostic utility of the CALLY index and CAR, multivariable logistic regression models were constructed using an *a priori* covariate selection strategy based on established clinical relevance and biological plausibility, rather than purely empirical automated stepwise selection. Age, sex, and serum creatinine were pre-specified as core adjustment covariates: age and sex represent fundamental non-modifiable demographic risk factors in acute PE, while serum creatinine accounts for baseline renal impairment, a major established confounder of both cardiovascular mortality and systemic inflammatory response. Other univariately significant parameters (such as blood urea, which collinearizes with creatinine, as well as lactate and LDH, which reflect downstream acute hypoperfusion and shock rather than baseline constitutional risk) were not included in the primary adjustment models to prevent over-adjustment, collinearity, and unnecessary sample size attrition due to missing values. To prevent collinearity between closely related composite metrics, CALLY and CAR were analyzed in separate models (Model 1 and Model 2).

All statistical tests were two-tailed, and a p-value of <0.05 was considered statistically significant.

#### Complete-case analysis statement

“A complete-case analysis was used for all statistical evaluations, and no data imputation was performed. Although the final analysis cohort comprised 399 patients, minor variations in sample sizes occurred across specific laboratory parameters and regression models due to missing values in the electronic health database. The exact number of patients (n) analyzed for each variable is explicitly stated in Table 3.”

#### Missing data handling

A structured missing-data strategy was applied without data imputation. Cohort-level exclusion was strictly applied to patients lacking essential clinical records or admission laboratory tests required for primary biomarker calculation, yielding the final cohort of 399 patients. Within this cohort, individual laboratory parameters were analyzed using an available-case approach, with the exact number of analyzed patients ($n$) reported for each variable in Table 3. Multivariable logistic regression models were executed as complete-case analyses restricted to patients with complete observations across all included covariates: Model 1 (categorical CALLY, *n* = 367; continuous log-CALLY, *n* = 366) and Model 2 (categorical CAR, *n* = 368; continuous log-CAR, *n* = 366).

## Results

A total of 399 patients diagnosed with acute pulmonary embolism were included in the study, of whom 311 (78.0%) survived and 88 (22.0%) died during hospitalization. The overall median age of the study population was 72.00 (IQR: 60.00–81.00) years, with non-survivors being significantly older than survivors [79.50 (IQR: 68.50–86.00) vs. 70.00 (IQR: 57.00–79.00) years, *p* < 0.001. Female predominance was observed in the overall cohort (63%), and the proportion of female patients was significantly higher in the non-survivor group (73% vs. 60%, p=0.042).

The CALLY index ratio, reflecting systemic inflammation and nutritional/immunological status, was significantly lower in non-survivors compared to survivors [0.71(0.19−1.77) vs. 1.56(0.54−5.02), p<0.001]. Regarding individual components of the CALLY index, non-survivors exhibited significantly reduced serum albumin levels [31.00(27.00−34.00) vs. 35.00(31.00−38.50) g/L, p<0.001] and absolute lymphocyte counts [1.26(0.87−1.79) vs. $1.57\;(1.10-2.23)\times 10^{9}/L$, p=0.003], alongside markedly elevated C-reactive protein (CRP) levels [74.45(18.63−129.09) vs. 32.60(11.56−82.00) mg/L, p=0.002]. Table 1 shows a comparison of the descriptive data.

**Table 1:** Baseline demographic, clinical, and laboratory characteristics of pulmonary embolism patients stratified by survival status.

Characteristic	Total Population (*N* = 399)	Survivors/Live (*n* = 311)	Non-Survivors/Ex (*n* = 88)	*p*-value
Age (years)	72.00(60.00−81.00)	70.00(57.00−79.00)	79.50(68.50−86.00)	<0.001
Sex, n (%)				0.042
Male	148(37%)	124(40%)	24(27%)	
Female	251(63%)	187(60%)	64(73%)	
CALLY Index Ratio	1.27(0.41−4.54)	1.56(0.54−5.02)	0.71(0.19−1.77)	<0.001
CRP/Albumine Ratio	1.15(0.37−2.87)	0.93(0.33−2.57)	2.37(0.68−4.42)	<0.001
Albumin (g/L)	34.00(30.00−38.00)	35.00(31.00−38.50)	31.00(27.00−34.00)	<0.001
CRP (mg/L)	38.88(12.60−93.40)	32.60(11.56−82.00)	74.45(18.63−129.09)	0.002
Absolute Lymphocyte ($10^{9}/L$)	1.48(1.01−2.10)	1.57(1.10−2.23)	1.26(0.87−1.79)	0.003
Absolute Neutrophil ($10^{9}/L$)	7.14(5.14−9.59)	6.82(4.92−9.19)	8.13(5.67−11.30)	0.009
WBC ($10^{3}/\mu L$)	9.90(7.60−12.80)	9.60(7.50−12.40)	11.00(8.10−14.10)	0.022
Fibrinogen (g/L)	2.39(2.20−4.14)	2.39(2.18−4.04)	2.40(2.20−4.48)	0.600
ESR/SEDİM (mm/h)	27.00(15.00−54.00)	29.00(17.00−53.50)	24.00(11.00−55.00)	0.200
Urea (mg/dL)	42.00(29.00−58.10)	39.00(28.00−52.00)	57.00(38.30−87.50)	<0.001
Creatinine (mg/dL)	0.85(0.66−1.07)	0.80(0.65−1.03)	0.95(0.76−1.36)	<0.001
Lactate/LAC (mmol/L)	1.90(1.30−2.70)	1.80(1.30−2.50)	2.30(1.50−3.60)	0.001
LDH (U/L)	298.50(241.00−373.00)	282.00(234.00−345.00)	356.00(293.00−445.00)	<0.001
Troponin (ng/L)	24.30(7.60−93.60)	23.00(6.80−93.30)	35.20(14.20−99.60)	0.038
AST (U/L)	26.00(19.00−39.00)	25.00(19.00−37.00)	31.00(22.00−44.00)	0.023
GGT (U/L)	35.00(21.00−64.00)	32.00(20.00−57.00)	39.00(23.00−94.00)	0.020
ALT (U/L)	22.00(14.00−41.00)	22.00(14.00−41.00)	21.00(12.00−38.00)	0.300
Glucose (mg/dL)	129.50(103.00−176.00)	124.00(102.00−159.00)	149.00(110.00−200.00)	0.004
Potassium (mEq/L)	4.35(3.97−4.70)	4.31(3.94−4.65)	4.53(4.09−4.93)	0.002
Sodium (mEq/L)	138.00(135.00−140.00)	138.00(135.00−140.00)	138.00(136.00−141.00)	0.900
Chloride (mEq/L)	104.00(101.00−106.00)	104.00(101.00−106.00)	103.00(101.00−105.00)	0.200
Hemoglobin (g/dL)	13.00(11.50−14.40)	13.20(11.70−14.40)	12.60(10.60−14.10)	0.044
Hematocrit (%)	39.20(34.90−42.90)	39.50(35.30−42.90)	37.50(32.50−43.60)	0.084
Platelet/PLT ($10^{3}/\mu L$)	230.00(176.00−298.00)	232.00(183.00−294.00)	220.00(166.00−310.00)	0.400
Absolute Monocyte ($10^{9}/L$)	0.75(0.55−1.00)	0.76(0.55−1.00)	0.74(0.53−1.02)	0.800
MCV (fL)	87.30(82.60−91.20)	87.40(82.80−91.10)	86.80(82.30−91.90)	>0.900
MPV (fL)	8.00(7.50−8.60)	8.00(7.50−8.60)	8.20(7.50−8.90)	0.200
Procalcitonin/PCT (%)	0.18(0.15−0.24)	0.19(0.15−0.24)	0.18(0.14−0.24)	0.500
$\;\mathrm{HC}O_{3}$ (mmol/L)	25.90(23.20−28.20)	26.00(23.50−28.30)	25.50(21.50−28.00)	0.200
$\;\mathrm{PC}O_{2}$ (mmHg)	41.80(36.80−47.40)	41.55(37.00−47.40)	42.20(36.20−47.60)	0.800
(mmHg)	30.40(22.90−41.00)	30.55(22.90−40.70)	28.60(23.40−41.70)	0.800

Receiver operating characteristic (ROC) curve analysis was conducted to evaluate and compare the predictive performance of the CALLY index and CAR for in-hospital mortality in patients with acute pulmonary embolism. Both biomarkers demonstrated statistically significant discriminative ability for predicting in-hospital mortality (p<0.001). The CALLY index yielded an area under the curve (AUC) of 0.655 (95%CI:0.584−0.726,p<0.001), whereas CAR achieved an AUC of 0.643 (95%CI:0.572−0.714,p<0.001). A pair-wise comparison of the two ROC curves using DeLong's test revealed no statistically significant difference between the AUC values of the two indices (p=0.582). According to the maximum Youden's J statistic, the optimal cutoff value for the CALLY index was determined as <1.17, demonstrating a sensitivity of 69.1%, specificity of 57.7%, positive predictive value (PPV) of 31.2%, and negative predictive value (NPV) of 86.4%. Conversely, the optimal cut-off value for CAR was >3.28, exhibiting a sensitivity of 42.0%, specificity of 83.2%, PPV of 40.7%, and NPV of 83.2%. While the CALLY index provided higher sensitivity for initial screening, CAR exhibited higher specificity, with both markers demonstrating negative predictive values above 83%. However, given the modest AUC values, these negative predictive values reflect the baseline survival prevalence and should not be considered sufficient to definitively rule out high-risk patients when used in isolation. The comparative ROC curve metrics are summarized in Table 2, and the corresponding ROC curves are illustrated in Figure 2.

**Table 2:** Receiver operating characteristic (ROC) curve metrics and predictive performance of the CALLY index and CAR for in-hospital mortality in patients with pulmonary embolism.

Biomarker	AUC	95% CI	*p*-value	Optimal Cut-off	Sensitivity	Specificity	PPV	NPV
CALLY Index	0.655	0.584−0.726	<0.001	<1.17	69.1%	57.7%	31.2%	86.4%
CAR (CRP/Albumin)	0.643	0.572−0.714	<0.001	>3.28	42.0%	83.2%	40.7%	83.2%

AUC, area under the curve; CI, confidence interval; PPV, positive predictive value; NPV, negative predictive value; CAR, C-reactive protein to albumin ratio; CALLY, C-reactive protein-albumin-lymphocyte ratio.

DeLong test comparison between CALLY and CAR AUCs: p=0.582.

**Figure 2:**
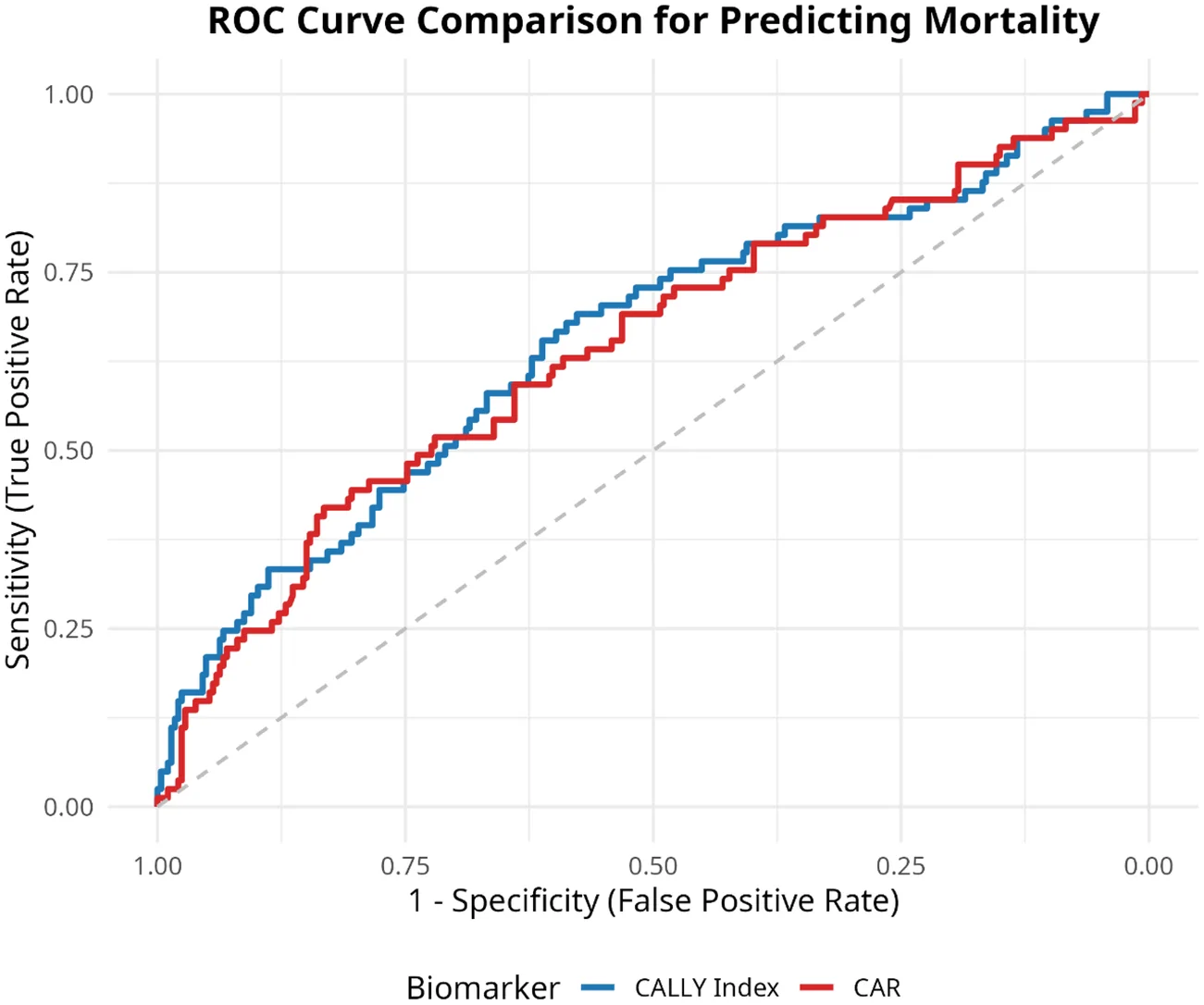
ROC curve comparison for predicting mortality.

Univariate and multivariate logistic regression analyses were conducted to evaluate the candidate clinical, inflammatory, and biochemical parameters as potential predictors of in-hospital mortality. Table 3 presents the results of the univariate and multivariate analyses.

**Table 3:** Univariate and multivariate logistic regression models identifying independent predictors of in-hospital mortality in patients with acute pulmonary embolism.

Predictor Variable	N	Univariate Analysis OR (95% CI)	*p*-value	Multivariate Model 1 (CALLY) aOR (95% CI)	Multivariate Model 2 (CAR) aOR (95% CI)	*p*-value (Multi)
Low CALLY Index (<1.17)^a^	367	3.05 (1.82–5.24)	<0.001	2.54 (1.46–4.50)	—	0.001
*Continuous log(CALLY)*	*366*	*0.74* (*0.63–0.88)*	*<0*.*001*	*0.75 (0.63–0.89)*	*—*	*0*.*001*
High CAR Ratio (>3.28)^a^	368	3.60 (2.10–6.18)	<0.001	—	3.02 (1.66–5.50)	<0.001
*Continuous log(CAR)*	*366*	*1.37* (*1.12–1.68)*	*0*.*002*	*—*	*1.39 (1.13–1.72)*	*0*.*002*
Age (per 1-year increase)	399	1.05 (1.03–1.07)	<0.001	1.04 (1.02–1.06)	1.04 (1.02–1.06)	<0.001
Sex (Female vs. Male)	399	1.77 (1.06–3.02)	0.032	1.68 (0.94–3.07)	1.80 (1.00–3.26)	0.050
Creatinine (mg/dL)	393	3.11 (1.86–5.46)	<0.001	2.71 (1.55–4.98)	2.56 (1.43–4.59)	0.002
Urea (mg/dL)	394	1.31 (1.20–1.43)	<0.001	—	—	—
LDH (per 10 U/L increase)	390	1.02 (1.01–1.03)	<0.001	—	—	—
Lactate (mmol/L)	351	1.17 (1.05–1.31)	0.005	—	—	—
Troponin (per 10 ng/L increase)	375	1.00 (1.00–1.00)	0.300	—	—	—
WBC (10³/*μ*L)	393	1.05 (1.00–1.11)	0.052	—	—	—

Values are presented as odds ratios (OR) or adjusted odds ratios (aOR) with 95% Confidence Intervals (CI). Model 1 evaluates the CALLY index (either categorical <1.17 or continuous log-transformed) adjusted for age, sex, and serum creatinine. Model 2 evaluates the CAR index (either categorical >3.28 or continuous log-transformed) adjusted for age, sex, and serum creatinine. ^a^Indicates secondary cut-off analysis. Analytic sample sizes vary across univariable evaluations based on available cases. Multivariable models represent complete-case analyses: Model 1 (CALLY <1.17, n = 367; log-CALLY, n = 366) and Model 2 (CAR >3.28, n = 368; log-CAR, n = 366). No imputation was performed.

## Discussion

In the present study, we evaluated the prognostic significance of the C-reactive protein-albumin-lymphocyte (CALLY) index in patients diagnosed with acute pulmonary embolism (PE) in an emergency department setting. Our findings demonstrate three major clinical insights: non-survivors exhibited significantly lower CALLY index values and higher CAR values compared to survivors; both a low CALLY index (<1.17) and a high CAR (>3.28) were identified as independent predictors of in-hospital mortality after adjusting for confounding factors such as age, sex, and renal function.

Acute pulmonary embolism triggers a complex cascade involving right ventricular strain, localized pulmonary vascular occlusion and ischemia, and a subsequent systemic inflammatory response. Inflammatory biomarkers, such as C-reactive protein (CRP), have long been recognized as indicators of systemic inflammation and hypercoagulability in acute PE. However, single inflammatory markers often lack the comprehensive scope needed to capture the complex pathophysiological states of critically ill patients. The CALLY index integrates three distinct biological dimensions: CRP reflects systemic inflammatory stress, albumin serves as a marker of nutritional status and negative acute-phase response, and absolute lymphocyte count represents host immune competence and cell-mediated stress response.

Factors affecting mortality in patients with pulmonary thromboembolism have been investigated in several studies. Age is a significant factor influencing mortality in patients with pulmonary thromboembolism; advanced age increases the risk of both early and late mortality (12, 13). In addition, comorbidities, especially heart failure, chronic obstructive pulmonary disease (COPD), hypertension, and diabetes, which are cardiovascular risk factors, increase the mortality risk (14–16). The presence of malignancy is another important factor that increases mortality in patients with pulmonary thromboembolism (12, 13). Among the laboratory findings, low PaO2 and high D-dimer levels stand out as indicators of poor prognosis (12, 16). Hemodynamic instability, right ventricular dysfunction, and elevated troponin levels are also associated with increased mortality (17, 18). Clinical scoring systems, particularly the Pulmonary Embolism Severity Index (PESI) and simplified PESI (sPESI), are considered reliable tools for predicting mortality in patients with pulmonary thromboembolism (15, 19, 20). Thrombolytic therapy has been found to be effective in reducing mortality, especially in patients with intermediate-risk pulmonary thromboembolism (19). Furthermore, in patients receiving thrombolytic therapy, factors such as the presence of shock, right ventricular hypokinesia, and elevated troponin levels further increase the mortality risk (18).

The CALLY index has emerged as an important biomarker for predicting mortality in various patient groups. In a study conducted on patients admitted to the intensive care unit with a diagnosis of decompensated heart failure, a low CALLY index was found to be inversely proportional to mortality, and it was stated that this index could serve as a guide for clinicians in determining the prognosis (21). In another study on patients with heart failure, the CALLY score was shown to be an independent factor in predicting in-hospital mortality, and lower CALLY scores were associated with a higher risk of death (22). Other studies have also demonstrated that the CALLY index is a significant prognostic parameter (23, 24). The C-reactive protein (CRP) ratio (CRP/Alb) has emerged as a strong biomarker for predicting mortality in various clinical scenarios. Reflecting systemic inflammation and nutritional status, this ratio is an effective tool for predicting early in-hospital mortality, particularly in elderly patients. For example, in a study conducted on elderly patients, a CRP/Alb ratio above 8 was identified as an independent predictor of 30-day mortality, and this ratio showed a strong association with predicting mortality within the first 7 days (25). Similarly, a study in patients with community-acquired pneumonia (CAP) demonstrated that the CRP/Alb ratio has a high discriminative power in predicting 30-day mortality, and a cutoff value above 0.77 provided high sensitivity and specificity (26). In patients with sepsis, a CRP/Alb ratio above 4.3 has been found to be an important indicator for predicting mortality (27).

Our results are consistent with recent studies highlighting the superiority of composite inflammatory-nutritional scores over single biomarkers in acute cardiovascular and thromboembolic diseases. Hypoalbuminemia, frequently observed in non-survivors in our cohort (31.00 vs. 35.00g/L, p<0.001), reflects both ongoing systemic capillary leakage and poor baseline nutritional reserve. Simultaneously, lymphopenia (1.26 vs. $1.57\times 10^{9}$/L, p=0.003) underscores the stress-induced immunosuppression mediated by hypercortisolemia and accelerated lymphocyte apoptosis in severe acute PE. When combined, a low CALLY index reflects an uncompensated inflammatory-nutritional-immunological collapse, explaining its robust association with nearly a 2.5-fold increase in adjusted in-hospital mortality risk (aOR=2.54, p=0.001).

When comparing the diagnostic and prognostic utility of the CALLY index with CAR, ROC curve analysis demonstrated comparable overall discriminative power, reflecting modest rather than strong discrimination (AUC=0.655 for CALLY vs. 0.643 for CAR, DeLong test *p* = 0.582). However, their performance profiles offer distinct clinical benefits: the CALLY index demonstrated higher sensitivity (69.1%), whereas CAR exhibited higher specificity (83.2%). While neither marker should be used in isolation to rule out high-risk patients due to their modest AUC values, their integration may support early risk stratification in the emergency department.

The C-reactive protein-to-albumin ratio (CAR) has increasingly emerged as a valuable prognostic marker reflecting systemic inflammation and nutritional compromise across various clinical settings, including older hospitalized patients, community-acquired pneumonia, and sepsis. However, disease-specific context within acute pulmonary embolism (PE) is essential. Recent multicenter data in acute pulmonary embolism reported a similarly modest discriminatory performance for CAR (AUC approximately 0.64), highlighting its utility when evaluated alongside inflammatory indices and clinical severity scores such as the simplified Pulmonary Embolism Severity Index (sPESI) [Çalışkan et al.]. Our findings corroborate these PE-specific observations (AUC = 0.643 for CAR), demonstrating that while single ratios like CAR possess modest overall discrimination, their integration within comprehensive clinical models offers valuable supportive information for early risk stratification (9)..

### Strengths and limitations

This study evaluated the prognostic associations of the CALLY index and CAR in a cohort of 399 patients with CTPA-confirmed acute pulmonary embolism presenting to the emergency department. Biomarkers were assessed using both cut-off thresholds and continuous log-transformed variables, and separate multivariable models were adjusted for age, sex, and serum creatinine.

Several limitations should be acknowledged. The retrospective, single-center design introduces potential selection bias and limits generalizability. Clinical variables needed to calculate PESI or sPESI, as well as echocardiographic right ventricular parameters (e.g., RV/LV ratio and TAPSE), were not consistently available and could not be included in multivariable models. Consequently, these findings do not establish that CALLY or CAR outperforms or adds incremental value to established clinical risk scores or imaging parameters. Biomarkers were measured only at emergency department presentation, precluding assessment of changes during hospitalization. Prospective multicenter studies are needed to validate the proposed CALLY cut-off of 1.17 and to assess whether incorporating these indices into established risk stratification approaches improves clinical decision-making.

## Conclusion

In conclusion, baseline CALLY index and CAR are readily available biomarkers independently associated with in-hospital mortality in patients with acute pulmonary embolism. However, because their discriminative performance is modest when used alone, they should not be used in isolation to rule out high-risk patients. They may provide supportive information as part of a comprehensive clinical risk assessment.

## Data Availability

The original contributions presented in the study are included in the article/Supplementary Material, further inquiries can be directed to the corresponding author.
