# Supplementary material for: Prognostic value of C-reactive protein-albumin-lymphocyte (CALLY) score and CRP-albumin ratio on mortality in patients diagnosed with pulmonary thromboembolism
Source: Front Med (Lausanne). 2026 Oct 26;13:1944670. doi: 10.3389/fmed.2026.1944670 (PMC13745728; doi:10.3389/fmed.2026.1944670)
Supplement: Supplementary file 1 [file Datasheet1.pdf]

STROBE STATEMENT CHECKLIST

Checklist of items that should be included in reports of *cohort/observational studies*

Title of Study: Prognostic Value of C-reactive protein-Albumin-Lymphocyte (CALLY) Score and CRP-Albumin Ratio on Mortality in Patients Diagnosed with Pulmonary Thromboembolism

Study Design: Retrospective Cohort / Observational Study (Single-Center, N=399)

Item No	STROBE Recommendation	Manuscript Context & Location
**TITLE AND ABSTRACT**		
1	**(a)** Indicate the study's design with a commonly used term in the title or the abstract.<br><br>**(b)** Provide in the abstract an informative and balanced summary of what was done and what was found.	Reported<br>**(a) Title & Abstract (Methods):** Identified as a "retrospective, single-center study".<br>**(b) Abstract:** Clearly summarizes background, objective, cohort size (N=399, 2021-2025), key findings (AUC values, ROC cut-offs, aOR values), and clinical conclusion.
**INTRODUCTION**		
2	**Background / Rationale:** Explain the scientific background and rationale for the investigation being reported.	Reported<br>**Introduction (Paragraphs 1-2):** Explains the acute PE mortality risk, physiological components of CALLY (CRP: inflammation, Albumin: nutrition, Lymphocyte: immunity), and rationale over CAR alone.
3	**Objectives:** State specific objectives, including any prespecified hypotheses.	Reported<br>**Introduction (Paragraph 2, last sentence):** States objective to examine the prognostic effect of initial emergency department CALLY index on mortality.
**METHODS**		
4	**Study Design:** Present key elements of study design early in the paper.	Reported<br>**Materials and Methods (Paragraph 1):** Retrospective observational single-center cohort study conducted at Elazığ Fethi Sekin City Hospital (01.01.2021 – 31.12.2025).
5	**Setting:** Describe the setting, locations, and relevant dates, including periods of recruitment, exposure, follow-up, and data collection.	Reported<br>**Materials and Methods (Paragraph 1):** Emergency Department setting; data extracted from Hospital Information Management System for patients diagnosed with PE between Jan 2021 and Dec 2025.
6	**Participants:** Give the eligibility criteria, and the sources and methods of selection of participants. Describe methods of follow-up.	Reported<br>**Materials and Methods (Paragraph 1):**<br>• *Inclusion:* Age >18 years, confirmed PE diagnosis via pulmonary CT angiography.<br>• *Exclusion:* Age <18 years, unconfirmed PE on clinical/imaging evaluation, incomplete electronic data.
7	**Variables:** Clearly define all outcomes, exposures, predictors, potential confounders, and effect modifiers. Give diagnostic criteria, if applicable.	Reported<br>**Materials and Methods (Paragraph 1-2) & Statistical Analysis:**<br>• *Primary Outcome:* In-hospital mortality (survivors vs non-survivors).<br>• *Primary Exposures:* CALLY score (Albumin × Lymphocyte / CRP) and CAR (CRP / Albumin).<br>• *Confounders/Adjustments:* Age, sex, serum creatinine.
8	**Data Sources / Measurement:** For each variable of interest, give sources of data and details of methods of assessment (measurement). Describe comparability of assessment methods if there is more than one group.	Reported<br>**Materials and Methods (Paragraph 1-2):** Extracted baseline demographic, clinical, and laboratory values at presentation from electronic health records (Hospital Information Management System).
9	**Bias:** Describe any efforts to address potential sources of bias.	Partially Reported<br>**Strengths & Limitations:** Retrospective single-center selection bias is acknowledged. Objective laboratory parameters used to minimize measurement bias. Multivariable logistic regression constructed to control for age, sex, and renal dysfunction.
10	**Study Size:** Explain how the study size was arrived at.	

Item No	STROBE Recommendation	Manuscript Context & Location
		Reported<br>**Materials and Methods / Results:** All consecutive eligible patients (N=399) presenting within the 5-year timeframe (2021–2025) meeting inclusion criteria were included (no formal sample size calculation reported).
11	**Quantitative Variables:** Explain how quantitative variables were handled in the analyses. If applicable, describe which groupings were chosen and why.	Reported<br>**Statistical Analysis:** Non-normally distributed variables expressed as Median (IQR). ROC curve & Youden's J index used to establish optimal cutoff values for CALLY (<1.17) and CAR (>3.28) for dichotomized regression and Kaplan-Meier analyses.
12	**Statistical Methods:**<br>**(a)** Describe all statistical methods, including those used to control for confounding.<br>**(b)** Describe any methods used to examine subgroups and interactions.<br>**(c)** Explain how missing data were addressed.<br>**(d)** Describe analytical methods taking account of sampling strategy.<br>**(e)** Describe any sensitivity analyses.	Reported<br>**Statistical Analysis:**<br>• **(a)** Mann-Whitney U, Chi-square/Fisher exact tests. ROC analysis with pROC, DeLong test. Univariate & multivariate logistic regression (Models 1 & 2 to prevent multicollinearity). Kaplan-Meier with log-rank tests.<br>• **(c)** Patients with incomplete data were excluded at screening.<br>• **(e)** Model 1 (CALLY) and Model 2 (CAR) evaluated separately to assess stability/multicollinearity.
**RESULTS**		
13	**Participants:**<br>**(a)** Report numbers of individuals at each stage of study.<br>**(b)** Give reasons for non-participation at each stage.<br>**(c)** Consider use of a flow diagram.	Reported<br>**Results (Paragraph 1) & Table 1:** Total N=399 analyzed (311 survivors [78.0%], 88 non-survivors [22.0%]). Exclusion criteria stated in Methods (age <18, missing data, unconfirmed PE).
14	**Descriptive Data:**<br>**(a)** Give characteristics of study participants and information on exposures and potential confounders.<br>**(b)** Indicate number of participants with missing data for each variable of interest.	Reported<br>**Table 1:** Detailed baseline demographic, inflammatory, clinical, and biochemical parameters for all 399 patients, stratified by survival status (median, IQR, p-values). Sample sizes per parameter reported in Table 3.
15	**Outcome Data:** Report numbers of outcome events or summary measures over time.	Reported<br>**Results & Figures 2–3:** 88 in-hospital deaths (22.0%). Time-to-event cumulative survival displayed via Kaplan-Meier curves with numbers at risk at 0, 100, 200, and 300 days.
16	**Main Results:**<br>**(a)** Give unadjusted estimates and, if applicable, confounder-adjusted estimates and their precision (e.g., 95% CI). Make clear which confounders were adjusted for and why they were included.<br>**(b)** Report category boundaries when continuous variables were categorized.<br>**(c)** If relevant, consider translating estimates of relative risk into absolute risk over a meaningful time period.	Reported<br>**Results & Tables 2–3:**<br>• **(a)** Unadjusted ORs and multivariable aORs with 95% CIs reported for Low CALLY (<1.17; aOR=2.54, 95% CI: 1.46-4.50, p=0.001) and High CAR (>3.28; aOR=3.02, 95% CI: 1.66-5.50, p<0.001), adjusted for age, sex, creatinine.<br>• **(b)** Cutoff thresholds (<1.17 and >3.28) established via Youden's J statistic.<br>• Diagnostic metrics reported: Sensitivity, Specificity, PPV, NPV.
17	**Other Analyses:** Report other analyses done—eg analyses of subgroups and interactions, and sensitivity analyses.	Reported<br>**Results (Table 2, Figures 1–3):** DeLong test comparing AUCs of CALLY (0.655) vs CAR (0.643) (p=0.582). Kaplan-Meier log-rank tests for CALLY (p<0.0001) and CAR (p=0.0001).
**DISCUSSION**		
18	**Key Results:** Summarise key results with reference to study objectives.	Reported<br>**Discussion (Paragraph 1):** Summarizes 3 main insights: lower CALLY/ higher CAR in non-survivors, independent prognostic role in multivariable models, and clear survival stratification on Kaplan-Meier analysis.
19	**Limitations:** Discuss limitations of the study, taking into account sources of potential bias or imprecision. Discuss both direction and magnitude of any potential bias.	Reported<br>**Discussion / Strengths and Limitations:** Highlights retrospective single-center design, lack of uniform sPESI/PESI scores and echocardiographic RV strain parameters, and absence of dynamic serial CALLY measurements.
20	**Interpretation:** Give a cautious overall interpretation of results considering objectives, limitations, multiplicity of	Reported<br>**Discussion (Paragraphs 2–6):** Contextualizes CALLY's biological plausibility (systemic inflammation, nutritional reserve, immune

Item No	STROBE Recommendation	Manuscript Context & Location
	analyses, results from similar studies, and other relevant evidence.	exhaustion) and compares findings with prior literature in PE, heart failure, and sepsis.
21	**Generalisability:** Discuss the generalisability (external validity) of the study results.	Reported<br>**Discussion & Conclusion:** Discusses emergency department applicability while recommending prospective multicenter validation before routine incorporation into guideline risk models (e.g., ESC PE classes).
**OTHER INFORMATION**		
22	**Funding:** Give the source of funding and the role of the funders for the present study and, if applicable, for the original study on which the present article is based.	Reported<br>**Ethics & Administrative Details:** Institutional study conducted at Elazığ Fethi Sekin City Hospital following local ethics committee approval.
